# The Hana Table Can Be Used for Successful and Safe Closed Reduction of Anterior and Posterior Total Hip Dislocations, Even in Prolonged Dislocation up to 36 Hours

**DOI:** 10.1016/j.artd.2026.101974

**Published:** 2026-03-10

**Authors:** Anhadh Jassal, Christian Princesa, Kenny Mai

**Affiliations:** a California Health Sciences University, Clovis, CA, USA; b St. George University School of Medicine, St. George’s, GD, USA; c Adventist Health, Hanford, CA, USA

**Keywords:** Total hip arthroplasty, Hip dislocation, Hana table, Closed reduction, Posterior dislocation, Anterior dislocation

## Abstract

Total hip arthroplasty (THA) dislocation remains a significant postoperative complication, and traditional closed reduction techniques can require substantial force with a risk of iatrogenic injury. We present 6 cases of postoperative THA dislocation, including 3 anterior and 3 posterior dislocations, successfully managed using the Hana fracture table. Five patients had primary anterior approach THA, and 1 underwent THA conversion via a posterior approach. One anterior dislocation presented 36 hours after occurrence. All reductions were performed under adequate sedation and muscle relaxation with controlled traction and fluoroscopic guidance. Closed reduction was successful in all cases without intraoperative or postoperative complications. At final follow-up, ranging from 6 months to 2 years, all hips remained stable without recurrent dislocation, supporting this technique as a safe and reproducible alternative.

## Introduction

Total hip arthroplasty (THA) is a widely performed procedure for the management of end-stage hip arthritis and femoral head osteonecrosis, offering significant improvements in pain relief and joint function. Despite advancements in implant design and surgical techniques, prosthetic hip dislocation remains a prevalent complication, with incidence rates ranging from 0.3% to 10% depending on various risk factors including, but not limited to, age, sex, body mass index, postsurgical patient activity, surgeon experience, and surgical approach [[1], [2], [3], [4], [5], [6], [7], [8]]. Closed reduction is the initial standard of care for managing early prosthetic hip dislocations, aiming to restore joint stability while minimizing the need for further surgical intervention [9,10].

Hip dislocations following THA can be categorized into anterior and posterior dislocations, each with distinct mechanisms and clinical implications. Posterior dislocations, which are the most common, typically occur when there is a combination of hip flexion, adduction, and internal rotation, leading to the femoral head being displaced posteriorly from the acetabulum [11]. This type of dislocation is more frequent with posterolateral approach due to the damage to posterior capsule and resulting weakness despite capsular repair [12]. In contrast, anterior dislocations are less common and typically occur when the hip is forced into extension, abduction, and external rotation. These dislocations are generally more challenging to manage due to the higher complexity of the reduction maneuver and are often associated with increased rates of complications such as femoral fracture and nerve injury [13].

Reduction techniques for prosthetic hip dislocations aim to restore the femoral head to its anatomical position within the acetabulum. Traditional closed reduction methods involve manipulation under anesthesia, often utilizing a combination of traction, flexion, extension, internal (or external rotation), and abduction (or adduction) to reposition the femoral head [9,14]. Traditional closed reduction techniques often require significant physical effort from the operating surgeon and may pose risks of periprosthetic fractures and soft tissue injury, particularly in cases where excessive force is needed [10,12]. Moreover, in settings such as the emergency department, inadequate assistance or the physical limitations of an aging surgeon workforce may present additional challenges in performing a successful reduction [15].

The Hana table, initially designed for anterior approach THA, has demonstrated utility in various orthopaedic procedures. Its precise positioning capabilities and controlled application of traction under fluoroscopic visualization offers a potential advantage in facilitating closed reductions of prosthetic hip dislocations with minimal effort from the surgeon and minimal risk of fracture with fluoroscopic guidance [7,16,17]. In this case report, we present a small series of successful closed total hip reductions for both anterior and posterior dislocations, including the anterior dislocation for at least 36 hours, with the use of a Hana table combined with sedation and muscle relaxant.

## Material and methods

From July 2023 to September 2024, 6 total hip dislocations, including 3 anterior and 3 posterior dislocations, were reduced using a Hana table. Five hip dislocations were following a primary THA using the anterior approach, and one dislocation required the removal of hardware and conversion to a THA using the posterolateral approach. Four dislocations occurred within 3 weeks postoperatively, primarily related to nonadherence to postoperative precautions, while 2 dislocations occurred approximately 2 months postoperatively following patient falls. None of the dislocations were associated with fractures, femoral stem subsidence, or changes in acetabular component positioning. Five dislocations were successfully reduced within 24 hours of the dislocation event. One anterior dislocation presented at least 36 hours after the initial event but was also successfully managed with closed reduction, without complications such as neurovascular injury or fracture. At the latest follow-up (ranging from 6 months to 2 years), all THAs remained stable and functioned well.

The Hana fracture table used in this series was manufactured by Mizuho OSI (Union City, CA, USA). While the Hana table was utilized in this study due to availability at our institution, the principles described are applicable to other orthopaedic traction or fracture tables capable of controlled longitudinal traction, limb positioning, and fluoroscopic access.

All patients were provided documented, informed verbal consent for publication of deidentified clinical details and images. The fluoroscopic imaging presented in this case series encompasses all intraoperative and periprocedural images available for review, consistent with the inherent limitations of a retrospective case series. Brief presentations of each patient will be stated below.

### Patient 1

Patient was an 84-year-old male who presented to the emergency department with a posterior hip dislocation 2 months after a left hemi hip arthroplasty secondary to a fall resulting in a femoral neck fracture. The patient had a successful surgery without acute complications. The hip dislocation event occurred 16 hours prior to presentation. This was the second dislocation since the hip arthroplasty. The first dislocation was reduced in a different facility’s emergency department. The patient demonstrated left hip flexion contracture to 90° and hip adduction across the body. The patient was consented, taken to the operating room, and the hip was reduced under anesthesia using the Hana fracture table. Anterior/posterior and lateral imaging confirmed the reduction. The patient’s hip was successfully evaluated at a 2-year follow-up appointment.

### Patient 2

Patient was a 73-year-old female who presented to the emergency department with an anterior hip dislocation 3 weeks post anterior approach left THA without complications. The patient dislocated her left hip when getting up from the seated position on a sofa 12 hours prior to presentation. A closed reduction was attempted in the emergency department but failed. The patient was consented, taken to the operating room, and the hip was reduced under anesthesia using the Hana fracture table. Anterior/posterior and lateral imaging confirmed the reduction. The hip remained stable at a follow-up appointment 1 year later.

### Patient 3

Patient was an 87-year-old female who presented to the emergency department with a posterior hip dislocation 5 weeks after an anterior approach left THA without complications. The patient was found by her family members with hip pain and bruising. The true time of dislocation was unknown but estimated to be greater than 36 hours prior to presentation. An unsuccessful closed reduction was attempted at a different facility’s emergency department. The patient was consented, taken to the operating room, and the hip was reduced under anesthesia using the Hana fracture table. Anterior/posterior and lateral imaging confirmed the reduction. The patient was discharged to a Skilled Nursing Facility. The hip remained stable at a follow-up appointment 18 months later.

### Patient 4

Patient was a 71-year-old female who presented to the emergency department with an anterior hip dislocation 3 weeks following a left anterior approach THA without complications. The patient noticed a pop in her hip, followed by severe pain when rolling out of bed 8 hours earlier. A closed reduction was attempted in the emergency department but was unsuccessful. The patient was consented, taken to the operating room, and the hip was reduced under anesthesia using the Hana fracture table. Anterior/posterior and lateral imaging confirmed the reduction. The hip remained stable at follow-up appointments 2 weeks and 6 months later.

### Patient 5

Patient was a 75-year-old female who presented to the emergency department with a posterior hip dislocation 1 week following a conversion to a left posterolateral approach THA. Five months prior, the patient underwent an open reduction and internal fixation that suffered from nonunion and cortical collapse requiring the conversion to a left THA. The patient suffered a posterior dislocation while Skilled Nursing Facility staff were changing her clothes 7 hours prior. The patient was consented, taken to the operating room, and the hip was reduced under anesthesia using the Hana fracture table. Anterior/posterior and lateral imaging confirmed the reduction. The hip remained stable at a follow-up appointment 6 months later.

### Patient 6

Patient was a 72-year-old male who presented to the emergency department with an anterior hip dislocation following a successful right anterior approach THA 15 days prior. The patient reported pain starting 8 hours ago but could not recall a specific event or trauma causing the dislocation. The patient was consented, taken to the operating room, and the hip was reduced under anesthesia using the Hana fracture table. Anterior/posterior and lateral imaging confirmed the reduction. The hip remained stable at a follow-up appointment 2 years later.

## Technique case 1—reduction for posterior dislocations

Following adequate sedation, the patient (Fig. a-c) was transferred to the Hana table and securely positioned in accordance with standard protocol. The operative leg was carefully padded and fixed in place to prevent soft tissue injury during the application of traction. A muscle relaxant was administered by the anesthesia provider to the maximum extent permitted by airway safety considerations.

**Figure 1:**
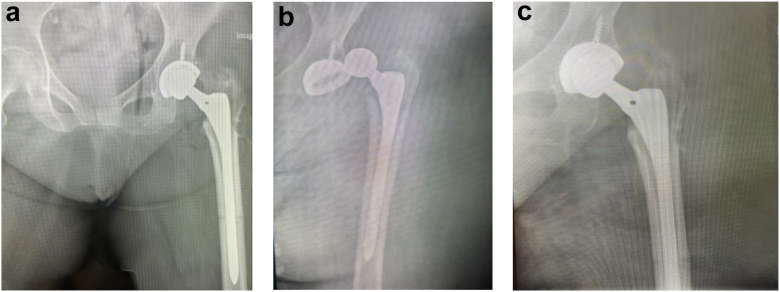
(a) Postoperative THA, (b) Posterior dislocation, (c) Post closed reduction using Hana Table.

A C-arm fluoroscope was positioned directly over the dislocated hip to provide continuous visualization. The dislocated hip was then gently internally rotated under fluoroscopic guidance to ensure that the femoral head was directed away from the patient’s body and not obstructed by the acetabular component during the reduction maneuver. Once appropriately aligned, the leg was locked into position.

Traction was then applied incrementally to the operative leg while monitoring the joint under fluoroscopy. This continued until the femoral head cleared the acetabular rim. In more challenging cases, the leg was maintained in traction for a few minutes following muscle relaxant administration, with periodic pauses to allow further muscle relaxation before resuming traction.

After the femoral head passed the rim and was positioned approximately at the level of the acetabular cup, the leg remained in traction and was then slowly externally rotated to guide the femoral head into the acetabular component. Gentle pressure on the greater trochanter was occasionally applied to assist in achieving complete reduction.

Once the femoral head was confirmed to be seated within the acetabular component under fluoroscopic visualization, traction was gradually released to allow the head to settle fully into its anatomical position. Anteroposterior and lateral fluoroscopic images were obtained to confirm appropriate alignment and to rule out periprosthetic fracture or component loosening.

The patient was then carefully transferred to a hospital bed and moved to the recovery area. Postreduction pelvic radiographs were taken to ensure the hip remained reduced during the transfer process.

## Technique case 2—reduction for anterior dislocation

**Figure 2:**
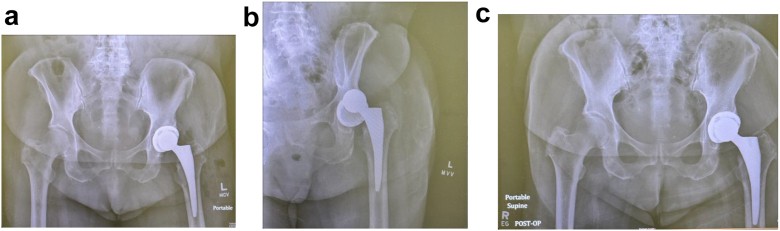
(a) Postoperative THA, (b) Anterior dislocation, (c) Post closed reduction using Hana Table.

In this study, anterior dislocations following THA performed via the anterior approach were reduced with minimal difficulty using the Hana table (Fig. 2a-c). After achieving adequate sedation and muscle relaxation, the patient was transferred to the Hana table and securely positioned according to standard protocol.

Subsequently, a C-arm fluoroscope was placed directly over the dislocated hip to provide continuous visualization throughout the procedure. Gradual traction was applied to the operative leg under fluoroscopic guidance. Because the femoral head in the anterior dislocation was displaced anterior to the acetabular component, the prosthesis was able to move inferiorly with traction without being impeded by the acetabular rim. Once the femoral head was adequately aligned with the center of the acetabular cup, the leg was locked into position.

The leg was then slowly internally rotated to facilitate the final positioning of the femoral head within the acetabulum. In some cases, gentle pressure on the greater trochanter helped guide the femoral head into the joint. Traction was then gradually reduced, allowing the femoral head to settle securely within the prosthetic cup.

Anteroposterior and lateral fluoroscopic views were obtained to confirm accurate reduction and to rule out any fractures or component displacement (Fig. 2c). The patient was subsequently transferred to a hospital bed and moved to recovery as per standard protocols. Postreduction pelvic radiographs were taken to ensure stability during transfer.

Due to anterior capsular attenuation or compromise resulting from the anterior surgical approach, the anterior dislocations were generally easier to reduce. Notably, one anterior dislocation that had remained unreduced for at least 36 hours was successfully managed without complications.

## Discussion

Amid rising healthcare costs, staffing reductions, and an aging orthopaedic workforce, cost-neutral innovations are essential to sustain and deliver high-quality orthopaedic care. In cases of dislocated THA, the traditional reduction techniques typically involve the provider climbing onto the bed to apply manual traction, while assistants stabilize the patient or provide countertraction. Availability of assistants late at night is often inadequate, especially in many rural hospitals such as where this study was conducted in Central California. As a result, cases are often delayed until the next day when adequate help is available. This occurrence has often delayed care to patients and increased burden on our hospitals due to increased length of stay. With Hana tables readily available in almost all hospitals, this study has explored techniques to reduce dislocated hips without the need of overwhelming strength and effort as in the case with traditional techniques. More importantly, closed reduction using a Hana table and x-ray imaging allows for the close visualization of components and adjustment of direction of reduction with minimal fracture risk. This procedure can be accomplished with one operating room nurse and operating room technician, which makes up the entire call crew at the facility at which this study was conducted; no first assists, physician assistants, or nurse practitioners are available on nights, weekends, or holidays at this facility.

Although this series utilized a Hana fracture table, the key advantages facilitating reduction were controlled longitudinal traction, stable limb positioning, and unobstructed fluoroscopic visualization. These features are not exclusive to a single manufacturer and may be achievable with other modern fracture or traction tables designed for orthopaedic procedures. Therefore, the described reduction technique may be reproducible in institutions equipped with alternative traction table systems, provided similar capabilities for incremental traction and imaging are available. Moreover, although the intraoperative fluoroscopic screenshots were not uniformly archived between patients, prereduction and postreduction images were shown to demonstrate dislocation pattern and successful restoration of prosthetic alignment following the described technique. In a future prospective study exploring this reduction technique, provisions should be put into place to provide relevant fluoroscopic imaging for each step of the reduction, potentially including continuous fluoroscopy for the most direct visualization of the technique.

This small case series, involving 6 total hip dislocations, provides encouraging reduction results with stable, well-functioning THA reductions with up to a 2-year follow-up. All 6 patients in this series underwent successful closed reductions without complications such as fracture, neurovascular injury, or component failure.

Of particular note was the successful closed reduction of one anterior dislocation after anterior approach THA. Due to transportation and social issues, the patient presented to the hospital over 36 hours from the time of dislocation. Traditionally, delayed dislocations are very difficult and often impossible due to soft tissue contracture and swelling. However, with general anesthesia, muscle relaxants, and slow traction with Hana table under fluoroscopic guidance, the hip was successfully reduced with minimal fracture risk.

Prior literature describing the use of fracture or traction tables for hip dislocation reduction remains very limited but supportive. Tremblay et al. reported successful and safe reduction of prosthetic hip dislocations using a fracture table, emphasizing improved control of traction forces and reduced physical demand on the surgeon [16]. Other reports describing fracture table assisted reductions of native and prosthetic hip dislocations similarly highlight improved fluoroscopic visualization and reduced risk of iatrogenic fracture when compared with traditional bedside techniques, but do not heavily focus on the technique itself. Nonetheless, our findings are consistent with these reports and further extend the literature by demonstrating feasibility across both anterior and posterior dislocations, including a delayed anterior dislocation.

Based on our experience, traditional closed reduction techniques may remain appropriate for early, uncomplicated dislocations with readily available personnel and successful initial attempts. However, fracture table assisted reduction should be considered in cases of failed emergency department reduction, delayed presentation, anterior dislocation, limited availability of assistants, or concern for excessive force required during manual reduction. In these scenarios, controlled traction under fluoroscopic guidance may reduce the risk of iatrogenic fracture, neurovascular injury, and procedural failure.

## Summary

Our early results provide some encouragement to deal with orthopaedic complications that often require strength and many assistants. During resource-limited periods, such as the COVID-19 pandemic, nights, weekends, and holidays, access to adequate assistance is often insufficient, particularly in smaller or rural hospitals. Minor adjustments in technique, such as performing closed reductions using the Hana table with fluoroscopic guidance, can facilitate high-quality care with fewer personnel and minimal risk of iatrogenic fracture. Therefore, closed reduction under fluoroscopic guidance, regardless of timing of presentation, has become our first-line approach to minimize the risk of iatrogenic fracture. Open reduction is reserved for cases in which closed reduction is unsuccessful.

## Conflicts of interest

The authors declare there are no conflicts of interest.

For full disclosure statements refer to https://doi.org/10.1016/j.artd.2026.101974.

## Informed patient consent

The author(s) confirm that written informed consent has been obtained from the involved patient(s) or if appropriate from the parent, guardian, power of attorney of the involved patient(s); and, they have given approval for this information to be published in this case report (series).

## CRediT authorship contribution statement

**Anhadh Jassal:** Writing – review & editing, Writing – original draft, Resources, Formal analysis, Data curation. **Christian Princesa:** Writing – review & editing, Writing – original draft, Validation. **Kenny Mai:** Writing – review & editing, Validation, Supervision, Project administration, Methodology, Investigation, Formal analysis, Conceptualization.

## Appendix A Supplementary data

Conflict of Interest Statement for Princesa

Conflict of Interest Statement for Mai

Conflict of Interest Statement for Jassal
