# Supplementary material for: The Hana Table Can Be Used for Successful and Safe Closed Reduction of Anterior and Posterior Total Hip Dislocations, Even in Prolonged Dislocation up to 36 Hours
Source: Arthroplast Today. 2026 Mar 10;38:101974. doi: 10.1016/j.artd.2026.101974 (PMC12993410; doi:10.1016/j.artd.2026.101974)
Supplement: Conflict of Interest Statement for Mai [file mmc2.pdf]

CONFLICT OF INTEREST STATEMENT

American Association of Hip and Knee Surgeons

(Adopted from the American Academy of Orthopaedic Surgeons disclosure statement)

The following form **must be filled out completely and submitted by each author (example, 6 authors, 6 forms). All items require a response. If there is no relevant disclosure for a given item, enter "*None.*"**

Manuscript Title The Hana Table Can Be Used for Successful and Safe Closed Reduction of Anterior and Posterior Total Hip Dislocations, Even in Prolonged Dislocation Up To 36 Hours

1. Royalties from a company or supplier (The following conflicts were disclosed)

   None

2. Speakers bureau/paid presentations for a company or supplier (The following conflicts were disclosed)

   None

3A. Paid employee for a company or supplier (The following conflicts were disclosed)

   None

3B. Paid consultant for a company or supplier (The following conflicts were disclosed)

   None

3C. Unpaid consultants for a company or supplier (The following conflicts were disclosed)

   None

4. Stock or stock options in a company or supplier (The following conflicts were disclosed)

   None

5. Research support from a company or supplier as a Principal Investigator (The following conflicts were disclosed)

   None

6. Other financial or material support from a company or supplier (The following conflicts were disclosed)

   None

7. Royalties, financial or material support from publishers (The following conflicts were disclosed)

   None

8. Medical/Orthopaedic publications editorial/governing board (The following conflicts were disclosed)

   None

9. Board member/committee appointments for a society (The following conflicts were disclosed)

   None

Each author must sign AND print or type his/her name, date and submit a separate form

In addition, one BLINDED Conflict of Interest form (no author names used) should be submitted per manuscript with all author disclosures.

Kenny Mai	[signature]	7/30/2025
Author Name (Print or Type)	Author Signature	Date
